# Efficacy and Tolerability of a Tri‐Acid Dermocosmetic Serum in Adults of Any Skin Phototype With Mild to Moderate Acne

**DOI:** 10.1111/jocd.71031

**Published:** 2026-07-06

**Authors:** Delphine Kerob, James Odeimi, Margot Broallier, Dominika Roziewska, Fook Chong, Jerry Tan

**Affiliations:** ^1^ La Roche‐Posay Laboratoire Dermatologique Levallois‐Perret France; ^2^ Eurofins Investigational Centre Gdansk Poland; ^3^ Insight Research Quatre Bornes Mauritius; ^4^ Western University, Department of Medicine and Windsor Clinical Research Inc Windsor Ontario Canada

**Keywords:** acne, dermocosmetic, quality of life, serum, skin aging

## Abstract

**Introduction and Objectives:**

Adult acne significantly impacts quality of life (QoL) and self‐esteem. Expert recommendations and guidelines recognize the role of dermocosmetics, either as a monotherapy for the mild to moderate forms, or as an adjunctive to acne medications in more severe forms. However, clinical data regarding the benefits of dermocosmetics in adult acne, with clinical visualization across all phototypes, is still lacking. This study assessed the benefit of an ultra‐concentrated tri‐acid complex serum (DC serum) in adult acne management in subjects of any skin phototype.

**Material and Methods:**

In an open‐label, multicentric study, 87 adult subjects of any phototype with a mean age of 25 ± 1 years, presenting mild to moderate acne (GEA 2–3), were recruited. DC serum was applied twice daily on the face for 168 days. Assessments comprised Global Evaluation of Acne (GEA) grading, submandibular acne scoring (in female participants only), and inflammatory lesion counts. Additionally, skin hydration levels, signs of cutaneous aging, quality of life (via the AI‐ADL scale), stigmatization (via PUSH‐D), local tolerance, and subject satisfaction rates were evaluated, alongside standardized clinical photography.

**Results:**

A significant (*p* < 0.0001) decrease in all lesion counts was observed starting 14 days. At Day 168, the inflammatory lesion count had decreased by 55%, that of non‐inflammatory lesions by 61%, and that of total lesions by 59%. The GEA score had significantly (*p* < 0.0001) decreased from Day 14, sustaining till Day 168 (−36%; 62% of subjects with an improvement). In women with submandibular acne, a 12%‐improvement at Day 14 (*n* = 63, *p* = 0.0078) and a 43%‐improvement at Day 168 (*n* = 56, *p* < 0.0001) were observed. Skin hydration had increased, and skin aging signs had significantly (*p* < 0.0001) improved at D168.

**Conclusion:**

DC serum is beneficial in mild to moderate adult acne across all phototypes, providing a continuous improvement of acne, skin hydration, and a reduction of early skin aging signs overtime.

## 1 Introduction

*Acne Vulgaris* is a chronic skin disease of the pilosebaceous follicles that affects 9.4% of the global population, ranking it eighth among skin conditions. It is characterized by non‐inflammatory lesions (open and closed comedones), inflammatory lesions (papules, pustules, nodules, and cysts) that can lead to marks and scarring, thus necessitating prolonged and persistent treatments [1]. While very frequently observed during adolescence, acne has an increasing impact and persistence in adult populations, especially among women [2]. In a study with 280 patients, 82.1% of the patients with adult acne were females [3].

Adult acne can be classified into three groups. “Persistent acne” which refers to acne that begins during adolescence and continues into adulthood. “Late‐onset acne” develops after the age of 25 years, and “Relapsing acne” describes acne that clears up after adolescence but then recurs in adulthood after initially appearing during puberty [4, 5]. Of these three types, persistent acne is currently the most common, accounting for up to 82% of adult acne cases [3, 6].

Adult acne has several key features, including pathophysiological factors such as hormonal status (particularly in women); clinical features such as submandibular acne (located along the U‐Zone), and more frequent inflammatory lesions that can develop into stubborn acne‐induced post‐inflammatory hyperpigmentation marks and scars. Moreover, it can last a lifetime and is influenced by the underlying skin aging process [7, 8, 9, 10].

Adult acne and its associated sequelae are strongly correlated with poor self‐image, depression, and anxiety, which negatively impact quality of life (QoL) [11]. A recent study highlights the profound psychosocial burden of acne sequelae, revealing that 35.5% of participants experienced scarring‐related limitations in their daily activities, while over a quarter of those with atrophic scars reported severe self‐consciousness, and 43.2% noted a detrimental impact on their interpersonal relationship [11].

Adult female acne is generally managed by various treatment approaches, including hormonal treatments (i.e., oral contraception) and off‐label use of spironolactone (an androgen receptor antagonist) [7, 8, 9, 10].

Expert recommendations and guidelines regarding the management of acne recognize the role of dermocosmetics more and more, either as a monotherapy for the milder to moderate forms or as an adjunctive to medications in more severe forms [12]. However, clinical data regarding the benefits of dermocosmetics in adult acne with clinical visualization across all phototypes remain scarce. The primary aim of this study was to assess the efficacy of a dermocosmetic serum (DC serum, Effaclar serum, La Roche‐Posay Laboratoire Dermatologique, France) in adult subjects with all skin phototypes and with mild to moderate acne. The secondary aim was to assess its anti‐aging and skin quality benefits. The DC serum contains a triple combination of exfoliating acids (salicylic, glycolic, and lipohydroxy acids) alongside soothing (niacinamide) and hydrating (glycerin) [13, 14, 15, 16, 17, 18, 19, 20].

## 2 Material and Methods

This multicentric, open‐label study was conducted at one investigational site in Mauritius and another in Poland between February 2023 and March 2024.

Due to its design, the study did not require ethics committee approval; no waiver was required. Nevertheless, the study conformed to all local legal requirements for the conduct of a clinical study and complied with the Principles of the Declaration of Helsinki. All subjects who participated in this study provided written informed consent prior to inclusion and provided written consent for their photographs to be taken during the course of this study.

The study included individuals aged 18 years or older, irrespective of gender or phototype. At least 10 subjects in each phototype group were to be recruited. Within each phototype group, at least 2 subjects were to be male. Five subjects had to have a facial GEA (Global Evaluation d'Acné [Global Acne Severity Scale]) grade 3 (moderate acne), and the remaining had to have a GEA grade 2 (mild acne). The GEA Scale is a global scale validated both on photographs and acne patients, which can be used either in clinical research or by dermatologists in their office [21]. Subjects were required to present with a minimum of 8 inflammatory and 20 non‐inflammatory facial acne lesions [21]. In women, the AFAST B (Adult Female Acne Scoring Tool; submandibular acne) score was used to assess submandibular acne severity [22]. Subjects who had taken oral isotretinoin within the last 6 months or had had any change in their hormonal treatment (women only) within the last 3 months or used a topical acne treatment within the last month were excluded.

After having cleansed their skin using their usual cleanser, subjects applied 4 to 5 drops of DC serum on the entire face while avoiding the eye area once a day, every evening, for 168 days. A provided sunscreen with an SPF 50+ (Anthelios UVmune 400, La Roche‐Posay Laboratoire Dermatologique, France) was to be applied once daily in the morning. Subjects were asked to avoid any excessive sun exposure during the study and not to make any changes to their usual skin hygiene or makeup habits.

Study visits took place at baseline/Day 0, Day 14, Day 28, and then every 28 days up to Day 168.

The primary endpoint was the total, inflammatory, and non‐inflammatory lesion count at all study visits assessed by the investigators.

Secondary endpoints included the assessment of the GEA score and of submandibular acne (if applicable) using AFAST B severity scoring, as well as different early skin aging signs detailed hereafter, instrumental measurements, subjects' QoL, and local tolerance.

Early skin aging signs (skin tone evenness, skin texture, smoothness, firmness, elasticity; all on a 10‐point scale with 0 = not marked to 9 = severely marked sign), presence of fine lines/wrinkles (from 0 = no fine lines/wrinkles to 9 = severe fine lines/wrinkles), overall mark severity and intensity including acne‐induced hyperpigmentation (AIH) (both on an 8‐point scale with 0 = no marks to 7 = many marks) were rated by both the investigators and subjects at each study visit.

Subjects' QoL was assessed using the AI‐ADL (Impact of Acne on the Daily Life of Adults) at baseline/Day 0, Day 84, and Day 168, and stigmatization using the PUSH‐D (Patient Unique Stigmatization Holistic tool in Dermatology) questionnaire at baseline/Day 0 and every 4 weeks until Day 168. Subjects also rated the cosmeticity of the DC serum using a self‐assessment questionnaire at Day 168 [23, 24].

Skin hydration was assessed using a corneometer (Corneometer, Courage + Khazaka electronic GmbH, Germany) at all visits. Photos were taken using a Skincam at baseline/Day 0, Day 84, and Day 168, and Colorface at all visits (both Qima LifeSciences, France).

Local tolerance to DC serum (erythema, dryness, desquamation, itching, stinging; all on a 5‐point scale from 0 = none to 4 = very severe) was assessed throughout the study, based on investigator assessments and subject‐reported side effects, including desquamation, itching, or stinging sensations.

Continuous data were summarized by each time point for the number of values, means, medians, standard deviations (SD), minimum, and maximum values. Categorical data, including QoL data, were summarized in frequency (N) and percentage (%) per time point. The total lesion count (sum of inflammatory and non‐inflammatory lesions) was calculated. The statistical efficacy analysis was performed on the intent‐to‐treat population. Missing data were carried forward.

Underlying assumptions were checked using the Shapiro–Wilk test (α = 0.01). In case of important deviations, data transformation or a non‐parametric approach (Wilcoxon signed rank tests) was performed. For all analyses, the type I error was set at α = 0.05 in a two‐tailed approach.

Excel 2016 (Microsoft, USA) and SAS 9.4 (SAS, USA) were used for the statistical analyses.

## 3 Results

A total of 87 subjects (69 women (79.3%) and 18 men) were included in this study. Their mean age was 25 ± 1.0 years (min: 18 years; max: 44 years); Among the participants, 43 had an oily skin type, while 44 had a combination skin type. Forty‐five subjects had a GEA grade 2, and 42 a GEA grade 3. Two subjects had a phototype I; phototype II and III were reported for 26 subjects each, 13 subjects had a phototype IV, 18 a phototype V, and 2 a phototype VI. Sixty‐nine females had submandibular acne.

Table 1 provides detailed baseline/Day 0 data about acne and skin aging signs assessed by the investigator, as well as results for skin hydration using corneometry.

**TABLE 1:** Baseline/day 0 subject, acne, and skin aging data (assessed by the investigator) as well as instrumental assessments.

Parameter		
**Inflammatory lesions**	Mean ± SD	10.8 ± 2.9
	Median	10.0
	Min; Max	8.0; 20.0
**Non‐inflammatory lesions**	Mean ± SD	29.6 ± 11.6
	Median	25.0
	Min; Max	15.0; 70.0
**Total lesions**	Mean ± SD	40.3 ± 12.4
	Median	37.0
	Min; Max	24.0; 78.0
**GEA grade**	Mean ± SD	2.5 ± 0.5
	Median Min; Max	2.0 2.0; 3.0
**AFAST b score**	Mean ± SD	1.1 ± 0.7
	Median	1.0
	Min; Max	0.0; 3.0
**Skin tone evenness**	Mean ± SD	4.4 ± 1.5
	Median	4.0
	Min; Max	0.0; 7.0
**Skin texture**	Mean ± SD	4.8 ± 1.3
	Median	5.0
	Min; Max	2.0; 9.0
**Skin smoothness**	Mean ± SD	6.7 ± 2.0
	Median	7.0
	Min; Max	2.0; 10.0
**Skin firmness**	Mean ± SD	8.1 ± 1.4
	Median	8.0
	Min; Max	4.0; 10.0
**Skin elasticity**	Mean ± SD	8.7 ± 1.1
	Median	9.0
	Min; Max	5.0; 10.0
**Fine lines**	Mean ± SD	1.5 ± 1.2
	Median	1.0
	Min; Max	0.0; 5.0
**Erythema**	Mean ± SD	1.2 ± 1.0
	Median	1.0
	Min; Max	0.0; 3.0
**Desquamation**	Mean ± SD	0.1 ± 0.3
	Median	0.0
	Min; Max	0.0; 2.0
**Itching**	Mean ± SD	0.2 ± 0.7
	Median	0.0
	Min; Max	0.0;4.0
**Stinging**	Mean ± SD	0.1 ± 0.4
	Median	0.0
	Min; Max	0.0; 2.0
**Overall disease severity**	Mean ± SD	3.6 ± 1.7
	Median	4.0
	Min; Max	0.0; 7.0
**Pigmentary intensity**	Mean ± SD	2.2 ± 1.0
	Median	2.0
	Min; Max	0.0; 4.0
**Skin hydration**	Mean ± SD	55.6 ± 11.0
	Median	55.5
	Min; Max	25.1; 78.6

**FIGURE 1:**
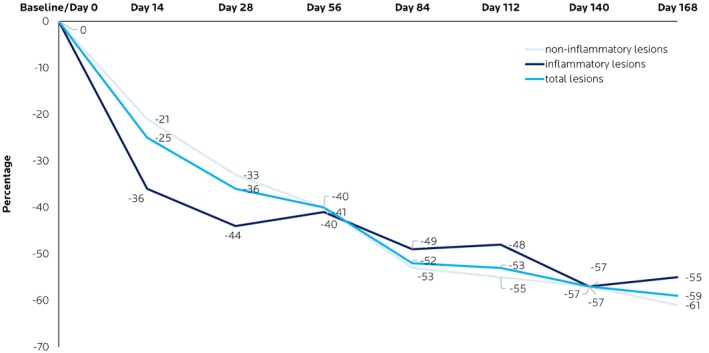
Evolution of the lesion count over time. The decrease from baseline of all lesion counts was significant (*p* < 0.0001) at all post‐baseline/Day 0 visits.

A significant (*p* < 0.0001) decrease in all lesion counts was observed as early as 14 days (inflammatory lesions: 36%, non‐inflammatory lesions: 21%, and total lesions: 25%). After 84 days, the decrease was 49% for inflammatory, 53% for non‐inflammatory, and 52% for total lesion count. At the end of the study (Day 168), the inflammatory lesion count had decreased by 55%, that of non‐inflammatory lesions by 61%, and that of the total lesions by 59% compared to baseline/Day 0. Figure 1 depicts the evolution of lesion counts over time.

**FIGURE 2:**
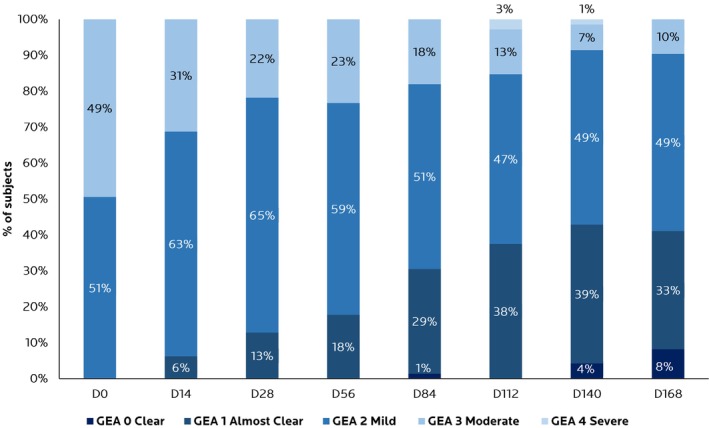
Evolution of the percentage of subjects with an improved GEA score: 41% of the subjects had their acne cleared/almost cleared at D168. Up to 60% of the subjects had their GEA grade improved by at least 1 grade at the end of the study.

**FIGURE 3:**
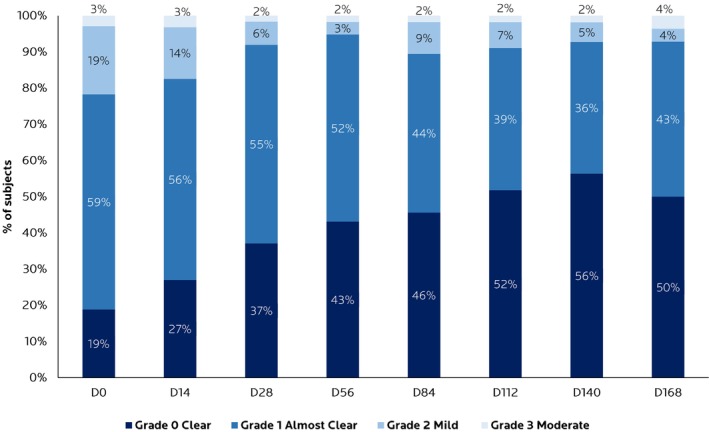
Percentage of female subjects with improved submandibular acne score. At the end of the study, 93% of the women had a cleared/almost cleared submandibular acne score. Almost 50% had their submandibular acne score improved by 1 grade.

The GEA score had significantly (*p* < 0.0001) decreased as early as Day 14 (−10%; 26% of subjects had improved), sustaining until Day 84 (−25%; 51% of subjects had improved; *p* < 0.0001) and Day 168 (−36%; 62% of subjects had improved). In women with submandibular acne (*n* = 69 at baseline), a 12%‐improvement at Day 14 (*n* = 63), a 37%‐improvement at Day 84 (*n* = 57) and a 43%‐improvement at Day 168 (*n* = 56) of the submandibular acne score were observed; improvement was significant (Day 14: *p* = 0.0078, Day 28 to Day 168: *p* < 0.0001). Figure 2 shows the evolution of the percentage of subjects with an improved GEA score from baseline/Day 0 to Day 168, and Figure 3 shows those with an enhanced submandibular acne score from baseline/Day 0 to Day 168.

**FIGURE 4:**
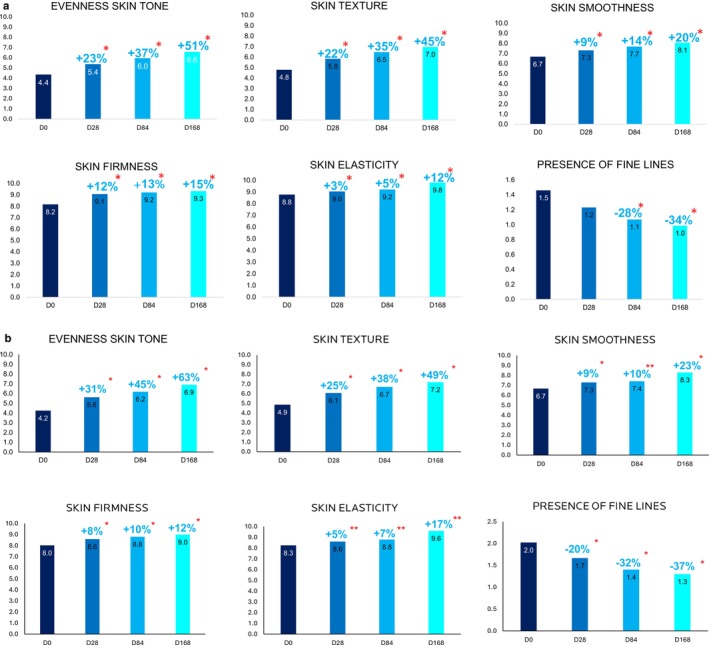
Evolution over time of skin aging signs.(a) Investigator assessment. *p < 0.0001. (b) Subject assessment. *p < 0.0001, **p < 0.05.

All early skin aging signs assessed had significantly (*p* < 0.0001) improved between baseline/Day 0 and Day 168, as shown in Figure 4a corresponding to the investigators' assessments, and Figure 4b to the subjects' assessments. According to the investigator, skin tone evenness improved by +51%, skin texture refinement by +45%, and fine line visibility by −34% at D168.

**FIGURE 5:**
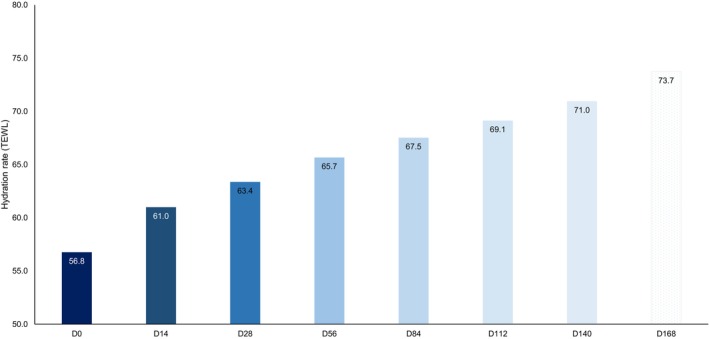
Evolution over time of skin hydration level. *p < 0.0001.

Skin hydration had significantly (*p* < 0.0001) improved as early as Day 14, and continued its significant (*p* < 0.0001) improvement until Day 168, showing a non‐drying effect of the tri‐acid DC serum (Figure 5).

According to the investigators, the overall acne‐induced hyperpigmentation (AIH) intensity and severity had significantly (*p* < 0.0001) improved at Day 168 (intensity: −29%; severity: −41%), with an onset as early as Day 28 (severity and intensity: −10% and −7%, respectively; *p* < 0.05). These results were paralleled by the subject ratings, providing similar significant (*p* < 0.0001) results on hyperpigmented lesions.

The subjects' QoL had significantly (*p* < 0.0001) improved after 84 days, with a 32% decrease of the AI‐ADL score from 32.8 points at baseline to 22.3 points, and by −45% (18.2 points) after 168 days; overall, the QoL of 81% of the subjects improved at Day 168.

The PUSH‐D score had significantly (*p* < 0.0001) decreased by 38% at Day 84 (16.2 points) from baseline/Day 0 (26.1 points) and by −61% (10.5%) at Day 168; the stigmatization score of 82% of the subjects had improved at Day 168.

The subjective benefits of the DC serum were highly regarded by participants: 89% perceived a visibly refined skin texture, 85% reported softer skin and less visible imperfections, and 84% observed a reduction in redness and improved skin comfort. In addition, the DC serum contributed to a visibly more even skin tone, as reported by 82% of subjects. It also resulted in less visible and tighter skin pores (80%) and reduced daytime skin shininess (72%). According to the subjects, the skin looked more radiant (81%) and firmer (77%) after having used DC serum for 168 days.

Local tolerance of DC serum was very good after 84 and 168 days, according to both subjects and the investigators, with almost no desquamation, itching, or stinging sensations reported at D168.

**FIGURE 6:**
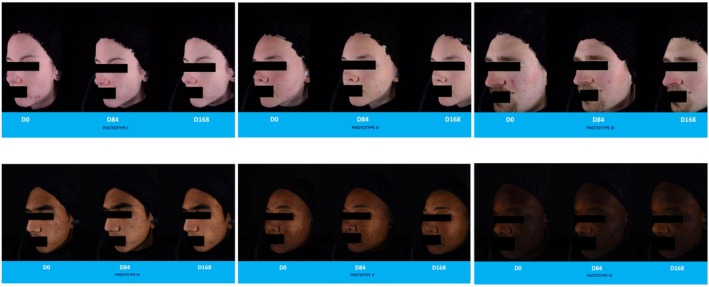
Examples for each phototype of subjects with improved acne after treatment with DC serum for 168 days.

Figure 6 provides examples of improvement for the best cases (men and women) for all 6 phototypes over time.

## 4 Discussion

Results obtained through this open‐label, uncontrolled study confirm previously shown outcomes that DC serum is associated with significant improvement of mild to moderate acne [25]. In the present study, DC serum, used once daily for 168 days, significantly (*p* < 0.0001) improved acne lesions, and as early as 14 days. Acne lesions had significantly (*p* < 0.0001) improved at Day 84 (−49% for inflammatory, −53% for non‐inflammatory, and −52% for total lesions), a commonly accepted treatment duration in acne management. As this study followed up subjects for 6 months (Day 168), further improvements in the inflammatory (−55%), non‐inflammatory (−61%), as well as the total lesion (−59%) count were observed. Moreover, the GEA and submandibular acne scores significantly (*p* < 0.0001) improved overtime. In addition to the improvement of acne and its sequelae, the study demonstrated that DC serum increases skin hydration, an important parameter in the preservation and restoration of the skin quality, which is frequently impaired after the use of medical acne treatments such as retinoids and/or benzoyl peroxide [26, 27]. In parallel, DC serum significantly (*p* < 0.0001) and gradually improved early skin aging signs, according to both investigators and subjects assessments. These primary and secondary outcomes of the present study may be considered due to the benefit of the unique combination of salicylic, glycolic, and lipohydroxy acids alongside niacinamide and glycerin [13, 14, 15, 16, 17, 18, 19, 20]. In total, 81% of the subjects stated that their QoL had improved; this was confirmed by a 45%‐decrease of the AI‐ADL score. The stigmatization score was also reduced by −61% at D168, with 82% of the subjects feeling less stigmatized at the end of the study. No tolerance concerns were reported by the investigators or by the subjects. Finally, the DC serum was highly appreciated by subjects regarding both cosmeticity and early aging signs enhancement.

In recent years, clinical trials have been more inclusive of patients, comparing the performance of treatments in different skin tones and types [28, 29, 30]. A large majority of studies have been conducted using pharmacological treatments, showing no efficacy differences between the different phototypes. Despite these findings, there is a growing recognition of phototype‐dependent variations in acne presentation. Therefore, further research across diverse populations is warranted to facilitate individualized care and potentially enhance treatment efficacy [31].

The present study provided evidence that dermocosmetics, in this case a serum containing a trio of peeling acids, significantly improve mild to moderate acne, as well as acne marks severity and intensity, and aging signs, regardless of the skin phototype in adult men and women.

These results underline a recently published consensus on recommendations for the use of dermocosmetics in acne, promoting the use of these products in milder to moderate forms of acne or in maintenance post‐acne medication, and as an adjunct to acne treatments [12]. Other treatment guidelines and recommendations for acne in adult subjects confirm this positioning [9, 32, 33, 34].

The tested DC serum was effective and well‐tolerated as monotherapy for mild to moderate forms of acne, especially during the first 2 weeks, in contrast to retinoid‐containing topicals, which usually induce skin dryness and irritation [35]. It has also been tested as a maintenance post‐acne treatments, showing its benefit in maintaining clinical efficacy and decreasing microcomedones formation, which is a key target to avoid acne relapses [25].

While skin aging may be considered less relevant in teenagers, early skin aging signs can be associated with adult acne and require adequate management, especially in adult women. Combining clinical benefit in acne with a “side clinical benefit” in skin aging in a single dermocosmetic serum may enhance adherence, satisfaction, and positively impact the subject's QoL and stigmatization, especially in women with acne [36, 37, 38, 39].

One main limit of this open‐label, single‐arm study is certainly the fact that the benefit of DC serum was only compared to baseline/D0 values before its use. Moreover, the study mainly recruited female subjects (79.3%). However, with a significant early and sustained treatment effect, and with adult acne mainly occurring in females, the results and distribution of the recruited subjects in the study confirm the clinical benefit of DC serum and reflect the distribution of acne in the adult population, which also parallels the observed gender distribution in another study [2, 3].

Despite these limitations, this study demonstrated that the tested dermocosmetic serum was effective and well‐tolerated in subjects of all phototypes presenting with mild to moderate acne. Significant efficacy was observed as early as 14 days, with continuous improvement extending up to 168 days. Furthermore, the serum significantly improved signs of skin aging while being highly appreciated.

## Author Contributions

M.B., J.O., and D.K. designed the study and supervised its conduct. Eurofins Poland and Insight Mauritius performed the study; J.O. wrote the manuscript and coordinated the different writting activities with the authors and the journal. All authors analyzed the data, reviewed, and approved the final manuscript.

## Funding

This study was supported by La Roche‐Posay Laboratoire Dermatologique, France.

## Ethics Statement

According to local legal requirements (Poland: European Regulations for the conduct of cosmetic studies: (CE) n° 1223/2009 and Mauritius: Clinical Trials Act 2011) for the conduct of this type of study using a commercially available cosmetic product, this study did not require approval from an ethics committee prior to its start. Nevertheless, the study conformed to all local legal requirements for the conduct of this type of study and complied with the Principles of the Declaration of Helsinki. Moreover, all subjects who participated in this study provided written informed consent prior to inclusion and provided written informed consent for the use of their photographs taken during the course of this study.

## Conflicts of Interest

M.B., J.O., and D.K. are employees of La Roche‐Posay Laboratoire Dermatologique. J.T. is a consultant and speaker for La Roche Posay, Laboratoire Dermatologique, France. F.C. and D.R. have no conflicts of interest to disclose.

## Data Availability

The data that support the results of this work are available from the corresponding author upon reasonable request.
